# Treatment of a Giant Haemangioma of the Liver With Kasabach-Merritt Syndrome by Orthotopic Liver Transplant

**DOI:** 10.1155/1997/10136

**Published:** 1997

**Authors:** J-H. Longeville, P. De La M. Hall, P. Dolan, A. W. Holt, P. E. Lillie, J. A. R. Williams, R. T. A. Padbury

**Affiliations:** 1 Department of Surgery Flinders Medical Centre Australia; 2 Department of Histopathology Flinders Medical Centre Australia; 3 Department of Anaesthesia Flinders Medical Centre Australia; 4 Department of Surgery Royal Adelaide Hospital Adelaide Australia

## Abstract

We describe a case of giant cavernous haemangioma of the liver with disseminated intravascular coagulopathy (Kasabach-Merritt syndrome) which was cured by orthotopic liver transplant.

A 47 year old man presented with bleeding and tender massive hepatomegaly after tooth extraction. Investigations showed disseminated intravascular coagulopathy and a giant hepatic haemangioma involving both lobes of the liver. Initial treatment failed to resolve the coagulopathy and liver resection was attempted. At laparotomy the turnout was unresectable and the only option for cure was to offer a liver transplantation. The orthotopic liver transplant was performed 20 days after initial laparotomy. Subsequently, all coagulation parameters returned to normal and the patient remains well after 12 months. Orthotopic liver transplant can be considered for giant hepatic haemangioma with Kasabach-Merritt syndrome when resection is necessary and a partial hepatectomy is not technically feasible.


*HPB Surgery*, 1997, Vol. 10, pp. 159–162
Reprints available directly from the publisher
Photocopying permitted by license only

© 1997 OPA (Overseas Publishers Association)
Amsterdam B.V. Published in The Netherlands
by Harwood Academic Publishers
Printed in India

### CASE REPORT
### Treatment of a Giant Haemangioma of The Liver With Kasabach-Merritt Syndrome By Orthotopic Liver Transplant a Case Report

J-H. LONGEVILLE*, P. DE LA M. HALL°, P. DOLAN*, A.W. HOLT†, P.E. LILLIE†, J.A.R. WILLIAMS# and R.T.A. PADBURY*

Department of *Surgery, °Histopathology, †Anaesthesia, Flinders Medical Centre, #Department of Surgery, Royal Adelaide Hospital, Adelaide, South Australia.

*(Received 10 August 1995)*

We describe a case of giant cavernous haemangioma of the liver with disseminated intravascular coagulopathy (Kasabach-Merritt syndrome) which was cured by orthotopic liver transplant.
A 47 year old man presented with bleeding and tender massive hepatomegaly after tooth extraction. Investigations showed disseminated intravascular coagulopathy and a giant hepatic haemangioma involving both lobes of the liver. Initial treatment failed to resolve the coagulopathy and liver resection was attempted. At laparotomy the tumour was unresectable and the only option for cure was to offer a liver transplantation. The orthotopic liver transplant was performed 20 days after initial laparotomy. Subsequently, all coagulation parameters returned to normal and the patient remains well after 12 months. Orthotopic liver transplant can be considered for giant hepatic haemangioma with Kasabach-Merritt syndrome when resection is necessary and a partial hepatectomy is not technically feasible.

KEY WORDS: Kasabach-Merritt syndrome   liver transplantation   haemangioma

### INTRODUCTION

Cavernous haemangioma is the most common benign tumour of the liver and is usually solitary and less than 4 cm in diameter.[1] Giant hepatic haemangioma is uncommon, rarely causes symptoms and requires no specific treatment when asymptomatic.[2]

The Kasabach-Merritt syndrome (KMS) describes the occurence of disseminated intravascular coagulopathy (DIC) with a large haemangioma and was first observed in association with a cutaneous haemangioma.[3] The natural course of the disease is characterised by a life-threatening coagulopathy. Treatment of this syndrome is often difficult, but resection of the tumour, when feasible, is usually curative.[4]

Only one case of orthotopic liver transplant (OLT) for an unresectable hepatic haemangioma has been previously described.[5] We report a second case of successful OLT for KMS with complete reversal of the coagulopathy postoperatively.

### CASE REPORT

A 47 year old man was referred to hospital for excessive bleeding after wisdom teeth extraction. His past and family history were unremarkable. On examination the patient was pale with obvious bleeding from the gum sockets and extensive bruising of the face. Abdominal examination revealed a tender massive hepatomegaly. Serial coagulation studies demonstrated a disseminated intravascular coagulopathy. Prothrombin time (PT) and activated partial thromboplastin time (APTT) were slightly prolonged at 17 sec (control: 11 sec) and 37 sec (control: 31 sec) respectively. The platelet count ($95 \times 10^3/\mu l$; *n*:

*Correspondence to*: Dr. RTA Padbury, Department of Surgery, Flinders Medical Centre, Bedford Park, 5042, Adelaide, South Australia.

150–450 × $10^3$/μl) and fibrinogen (0.6 gr/l; *n*: 2–4.5 gr/1) were low and the XL-fibrinogen degradation products (XL-FDP) were significantly elevated at 4–8 gr/1 (*n*<0.25 gr/1). The Euglobin lysis time was greater than 90 mins. The antithrombin 3 plasma level was low at 69% (Normal range: 79%–120%.). The leucocyte and red blood cell counts were within the normal range.

Abdominal CT scan revealed an exophytic tumour, 25 cm in diameter, replacing the left lobe and invading the right lobe of the liver, and extending inferiorly into the pelvis Figure 1. Following contrast, the periphery of the mass strongly enhanced and delayed scans showed filling from the periphery to the centre of the mass. A second 4 cm tumour suggestive of haemangioma was also seen in the posterior sector of the right lobe. A 99mTc red cells scan of liver confirmed the diagnosis of haemangioma.

The patient was treated with low dose i.v. heparin (500 IU/h) and received multiple cryoprecipitate transfusions. Packing of the oral bleeding areas assisted with the control of the haemorrhage. Heparin therapy and cryoprecipitate transfusion resulted in a slight increase of fibrinogen levels and control of the DIC, but failed to improve the platelet count. Continuing heparin administration was necessary to maintain fibrinogen levels. To correct the coagulopathy, the decision was taken to proceed to surgical removal of the larger haemangioma.

The heparin infusion was ceased 2 hours prior to laparotomy and in total 40 units of cryoprecipitate had been administered preoperatively. During initial laparotomy, the tumour was found to be extending extensively towards the right lobe of the liver (which was compressed by the large bulk of the tumour) with an absence of a clear peritumoral capsule on the intraoperative ultrasound (and subsequently confirmed by histopathology). Furthermore, the ultrasound appearances suggested that the margins of the lesion surrounded the right hepatic vein, thereby rendering resection impossible. Neither the preoperative CT scan nor the intraoperative ultrasound identified a right subhepatic vein, the presence of which may have rendered the haemangioma resectable.

After the initial surgical assessment, it was considered that the only therapeutic option which might achieve cure was a liver transplantation.

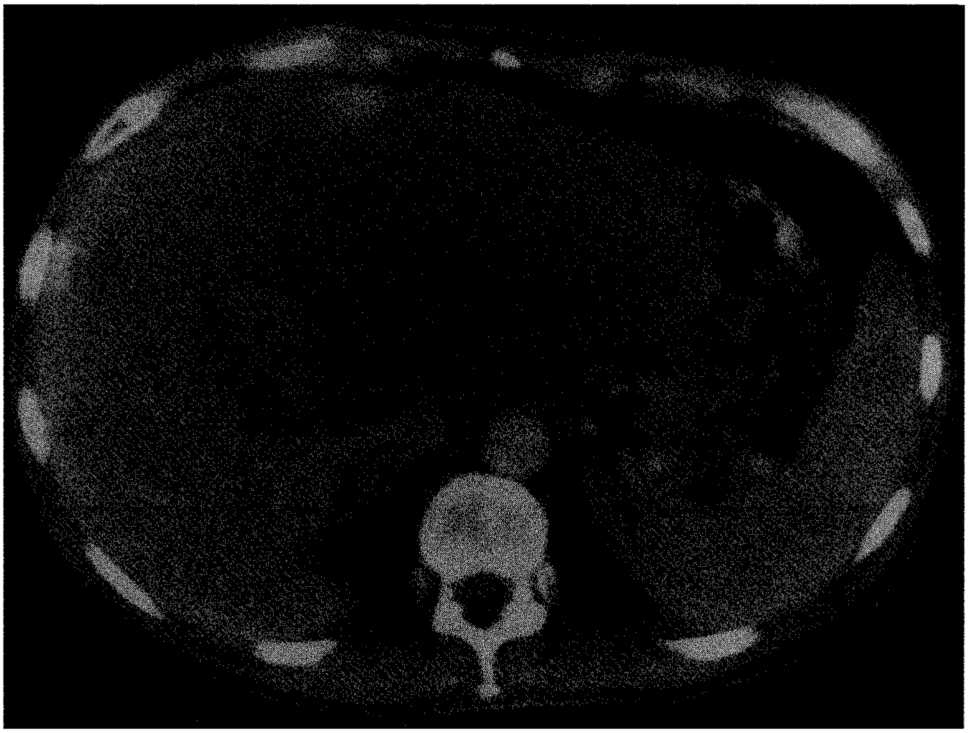

**Figure 1** CT scan section demonstrating the close relationship between the cavernous haemangioma and the right hepatic vein (Arrowhead).

While awaiting liver transplantation the DIC persisted and heparinisation remained necessary. OLT was performed 20 days after the exploratory laparotomy. The heparin infusion was ceased approximately 4 hours prior to the induction of anaesthesia. Following anaesthetic induction an aprotinin (Trasylol®, Bayer, Pymble, Australia) infusion was commenced ($2\times10^6$ KI units loading dose followed by a $5\times10^5$ KI units/hour continuous infusion). A femoral bypass cannula was inserted percutaneously prior to commencement of abdominal procedure. Rapid clotting of the femoral cannula after insertion precluded the use of bypass and it was decided to cease the aprotinin infusion.

The OLT procedure was a standard piggy-back technique. Total operating time was 9 hours and the total ischaemic time of the donor liver was 12 hours. The patient received a total of 14 units of blood, 16 units of fresh frozen plasma, 17 units of platelets and 2 units of cryoprecipitate during the procedure.

The recipient hepatectomy was a difficult procedure because of the bulk and weight of the tumour. Access to the hepatic pedicle was restricted and to gain exposure to control haemorrhage the pedicle was mass clamped and divided.

With further manipulation the haemangioma progressively emptied, became significantly smaller, and the surgical exposure improved.

The patient did not receive heparin nor aprotinin infusions postoperatively. The early postoperative course was marked by an intraabdominal haemorrhage. The patient was returned to theatre and the only site of haemmorrhage was from divided ligaments on the right side of the donor liver. The subsequent recovery was uneventful. Complete correction of coagulation parameters was achieved within 24 hours post-transplantation. The PT and APTT were normal within 12 hours of removal of the haemangioma. There was a dramatic fall in the XL-FDP levels within 24 hours. The patient remains well and symptomless 12 months after the procedure.

The liver weighted 3570 gr; the entire left lobe and a portion of the adjacent right lobe were replaced by a giant cavernous haemangioma Figure 2. A separate subcapsular haemangioma, maximum diameter 35 mm, was also present in the right lobe. The main tumour was poorly encapsulated and sections from the right lobe of the liver adjacent to the tumour showed focal areas of cavernous haemangioma intermingled with normal liver Figure 3. Multiple old and recent thrombi were seen within the haemangioma.

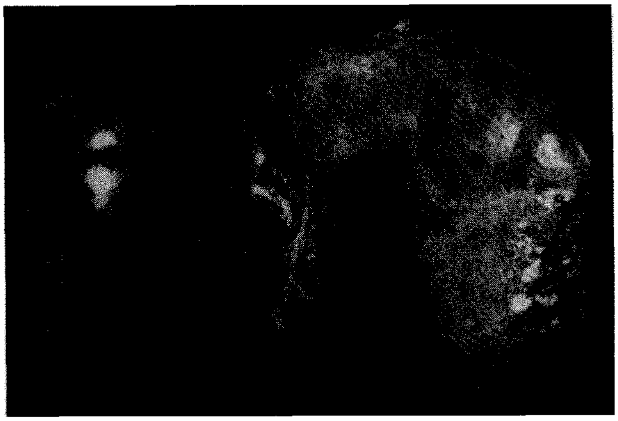

**Figure 2** "Native" liver showing complete replacement of the left lobe by a giant haemangioma. The tumour also involves the medial aspect of the right lobe.

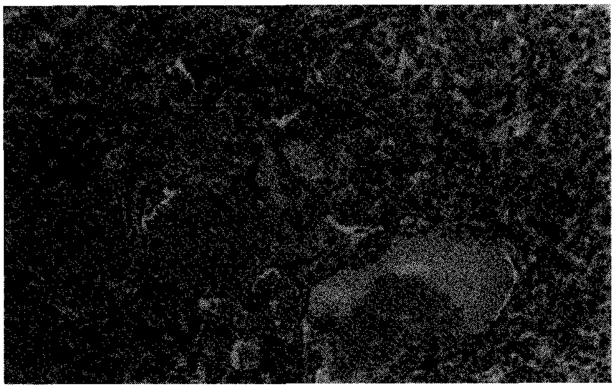

**Figure 3** Section of the right lobe of liver, taken some distance from the main tumour mass, showing one of the separate small cavernous haemangioma. Normal hepatic parenchyma surrounds the collection of thin-walled blood vessels. H&E × 110.

### DISCUSSION

The vast majority of hepatic cavernous haemangiomas remain asymptomatic and are discovered incidentally.[6] Rarely, a large haemangioma may cause right upper quadrant pain, but the potential for rupture is negligible and does not in itself constitute an indication for resection.[7] Malignant transformation is unknown.

KMS is a consumptive coagulopathy described originally in association with cutaneous haemangiomas in children.[8] This coagulopathy is thought to be related to a localised form of intravascular coagulation within the haemangioma. KMS associated with hepatic haemangiomas is rare, but well-known, and raises a number of management problems.

There have been several reports of the use of low dose heparin infusion, cryoprecipitate and aminocaproic acid to control the intravascular coagulopathy.[9,10] In our case heparinisation decreased the DIC but failed to cure completely the coagulopathy. Removal of the tumour was required, and partial hepatectomy, if technically feasible, was considered a superior option to OLT in the first instance. The presence of the second, considerably smaller, haemangioma in the right lobe of the liver was not considered a contraindication to attempt partial hepatectomy as KMS does not occur in association with small haemangiomas. There have been a variety of surgical techniques advocated to resect hepatic cavernous haemangiomas. Most authors advocate formal liver resection.

Alternatively, there have been reports of "enucleation" of the tumour following a cleavage plane separating the fibrous capsule of the tumour and the normal liver parenchyma.[11] In our case, the extension of the tumour towards the right liver with involvement of the right hepatic vein, and the absence of a peritumoral capsule, precluded the possibility of limited hepatic resection or enucleation during the first laparotomy.

The presence of an ongoing coagulopathy raised technical problems during the OLT. Because of the improvement of the DIC and a concern about intraoperative and postoperative bleeding the low dose heparin infusion was stopped before the OLT. This heparin-free period was associated with decreased fibrinogen and increased FDP. The rapid clotting of the femoral cannula at the beginning of the procedure, in conjunction with the preoperative abnormal antithrombin levels, raised further concern about concomitant hypercoagulability and the aprotinin infusion was ceased. In retrospect, it may have been optimal to continue heparin until the end of the hepatectomy to control the DIC and to prevent the clotting of the venous accesses.

OLT can be performed safely in giant haemangiomata complicated by KMS when resection is necessary but technically impossible.

### REFERENCES

1 MacSween, R.N.M., Anthony, P.P., Scheuer, P.J., Burt, A.D. and Portmann, B.C. (1994) Pathology of the liver. 3rd ed. Churchill Livingstone.
2. Adam, Y.G., Huvos, A.G. and Fortner, J.G. (1970) Giant hemangiomas of the liver. *Annals of Surgery*, **172**, 239–45.
3. Kasabach, H.H., and Merritt, K.K. (1940) Capillary hemangioma with extensive purpura: report of a case. *American Journal of Diseases of Children*., **59**, 1063–70.
4. Watzke, H.H., Linkesch, W. and Hay U. (1989) Giant hemangioma of the liver (Kasabach-Merritt syndrome): Sucessful suppression of intravascular coagulation permitting surgical removal. *Journal of Clinical Gastroenterology*., **11**, 347–50.
5. Klompmaker, I.J., Sloof, M.J.H., van der Meer, J., de Jonk, G.M.Th, de Bruijn, K.M. and Bams, J.L. (1989) Orthotopic liver transplantation in a patient with a giant cavernous hemangioma of the liver and Kasabach-Merritt syndrome. *Transplantation*., **48**, 149–51.
6. Tait, N., Richardson, A.J., Muguti, G., and Little, J.M. (1992) Hepatic cavernous hemangioma: A 10 year review. *The Australian and New Zealand Journal of Surgery*., **62**, 521–24.
7. Schwartz, S.I., and Cowles Husser, W. Cavernous hemangioma of the liver. (1987) A single institution report of 16 resections. *Annals of Surgery*., **205**, 456–63.
8. Inglefield, J.T., Tisdale, P.D.and Fairchild, J.P. (1961) A case of hemangioma with thrombocytopenia in the newborn infant treated by total excision. *Journal of Pediatrics*., **59**, 138–142.
9. Warrell, R.P., and Kempin, S.J. (1985) Treatment of severe coagulopathy in Kasabach-Merritt syndrome with aminocaproic acid and cryoprecipitate. *New England Journal of Medicine*., **313**, 309–312.
10. Stahl, R.L., Henderson, J.M., Hooks, M.A., Martin, L.G. and Duncan, A. (1991) Therapy of the Kasabach-Merritt syndrome with cryoprecipitate plus intra-arterial thrombin and aminocaproic acid. *American Journal of Hematology*., **36**, 272–74.
11. Alper A, Ariogul O, Emre A, Uras, A. and Okten A. (1988) Treatment of liver hemangiomas by enucleation. *Archives of Surgery*., **123**, 660–61.

